# Assessment of Epinephrine and Norepinephrine in Gastric Carcinoma

**DOI:** 10.3390/ijms22042042

**Published:** 2021-02-18

**Authors:** Alina Maria Mehedințeanu, Veronica Sfredel, Puiu Olivian Stovicek, Michael Schenker, Georgică Costinel Târtea, Octavian Istrătoaie, Ana-Maria Ciurea, Cristin Constantin Vere

**Affiliations:** 1 Department of Oncology, University of Medicine and Pharmacy of Craiova, 200349 Craiova, Romania; alina.maria591@gmail.com (A.M.M.); michael.schenker@umfcv.ro (M.S.); amciurea14@gmail.com (A.-M.C.); 2 Department of Physiology, University of Medicine and Pharmacy of Craiova, 200349 Craiova, Romania; veronica.sfredel@umfcv.ro; 3 Department of Pharmacology, Faculty of Nursing, Târgu Jiu Subsidiary, Titu Maiorescu University, 04317 Bucharest, Romania; olivian_sfn@yahoo.com; 4 Department of Cardiology, University of Medicine and Pharmacy of Craiova, 200349 Craiova, Romania; 5 Department of Gastroenterology, University of Medicine and Pharmacy of Craiova, 200349 Craiova, Romania; cristin.vere@umfcv.ro

**Keywords:** gastric carcinoma, norepinephrine transporter, plasma free metanephrines and normetanephrines

## Abstract

The aim of our study was to assess the sympathetic nervous system’s involvement in the evolution of gastric carcinoma in patients by analyzing the mediators of this system (epinephrine and norepinephrine), as well as by analyzing the histological expression of the norepinephrine transporter (NET). We conducted an observational study including 91 patients diagnosed with gastric carcinoma and an additional 200 patients without cancer between November 2017 and October 2018. We set the primary endpoint as mortality from any cause in the first two years after enrolment in the study. The patients were monitored by a 24-h Holter electrocardiogram (ECG) to assess sympathetic or parasympathetic predominance. Blood was also collected from the patients to measure plasma free metanephrine (Meta) and normetanephrine (N-Meta), and tumor histological samples were collected for the analysis of NET expression. All of this was performed prior to the application of any antineoplastic therapy. Each patient was monitored for two years. We found higher heart rates in patients with gastric carcinoma than those without cancer. Regarding Meta and N-Meta, elevated levels were recorded in the patients with gastric carcinoma, correlating with the degree of tumor differentiation and other negative prognostic factors such as tumor invasion, lymph node metastasis, and distant metastases. Elevated Meta and N-Meta was also associated with a poor survival rate. All these data suggest that the predominance of the sympathetic nervous system’s activity predicts increased gastric carcinoma severity.

## 1. Introduction

Gastric cancer is a malignant disease with a high degree of lethality; according to Globocan 2018, it ranks fifth in terms of the incidence of malignancies, with 1,033,701 cases annually (5.7%). It is also the third leading cause of cancer mortality, with an annual death toll of approximately 782,685 (8.2%) [1]. This condition is much more common among men, occupying third place for the total number of neoplasms, whereas this condition is ranked in fifth place regarding incidence for women [1]. The factors leading to this disease have not been identified exactly, but strong correlations have been found between its occurrence and diet (i.e., a diet rich in salty and smoked foods), *Helicobacter pylori* infection, vitamin deficiency, a low consumption of fruits and vegetables, smoking, a family history of gastric cancer, stress, and long-term stomach inflammation [2].

The evolution of gastric cancer is unpredictable, and because of the extensive nature of the diagnostic methods, as well as diagnosis often occurring in advanced stages of the disease, the disease only becomes symptomatic in the advanced stages for the vast majority of patients [3]. The involvement of the autonomic nervous system in the development and evolution of gastric cancer has not been fully elucidated, but numerous studies have shown that there is a close relationship. Not only does the autonomic nervous system innervate the digestive tract but the tumor cells secrete growth factors and exhibit elevated levels of catecholamines and various receptors [4,5,6]. The involvement of the vegetative nervous system was first demonstrated by Batsakis approximately 30 years ago when he described the presence of nerves located in the vicinity of human epithelial carcinomas, such as gastric, head and neck, or prostate cancers [7,8]. These nerves have been described as directly involved in metastatic dissemination through a process called perineural invasion (PNI) in which neoplastic cancer cells are able to invade and migrate into, around, and through the nerves, with PNI frequently being associated with poor clinical results [9].

In this study, we wanted to evaluate the involvement of the sympathetic nervous system in the evolution of patients with gastric carcinoma by analyzing the mediators of this system (epinephrine and norepinephrine) as well as the histological expression of the norepinephrine transporter (NET). The norepinephrine transporter is a monoamine transporter responsible for capturing extracellular norepinephrine (N-Meta), also known as noradrenaline. The latter has an inhibitory role in the gastrointestinal tract. This is also true for epinephrine, which mainly enters the gastrointestinal tract through the bloodstream after being secreted by the adrenal medulla directly into the circulation. It is known that this transporter is also involved in the uptake of extracellular dopamine; the reuptake of the two neurotransmitters plays an important role in regulating their concentrations in the synaptic terminals [10,11,12,13].

In order to obtain homogeneous data, we chose to analyze the influence of epinephrine and norepinephrine only on gastric tumors because of the physiological particularities of the stomach’s innervation. It is necessary to mention that the stomach is much more dependent on extrinsic neural inputs, represented by nuclei located in the caudal brainstem, from which sympathetic and parasympathetic pathways start or are controlled. In contrast to the stomach, the small and large intestines have a high degree of independent neuronal control and can function even if they lack extrinsic neural inputs [14].

## 2. Results

### 2.1. Assessment of the Heart Rates of the Patients Included in the Study

**Figure 1:**
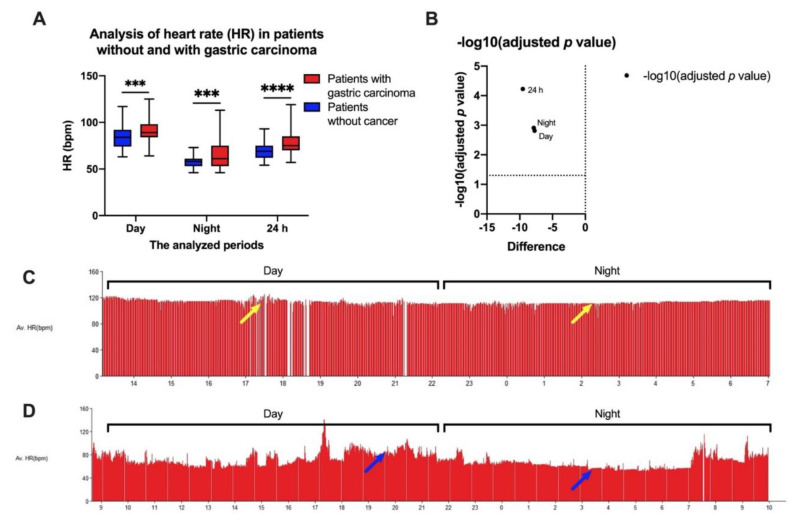
Evaluation of heart rates (HRs) by Holter electrocardiogram (ECG) of the patients included in the study. (**A**) Minimum, maximum, and average HRs in patients with gastric carcinoma and patients without cancer, with (**B**) a volcano association plot. (**C**) Representative histogram of HR for a 24 h period of a patient with gastric carcinoma; approximately the same HRs can be observed during the day and night (yellow arrows). (**D**) Representative histogram of HR for a 24 h period of a cancer-free patient; it was higher during the day (as normal) and lower at night (blue arrows). Av HR, atrioventricular heart rate; bpm, beats per minute. Student’s *t*-test, *** *p* < 0.001 and **** *p* < 0.0001.

To assess the predominance of the sympathetic or parasympathetic autonomic nervous system, we evaluated 91 patients suffering from gastric cancer using a Holter electrocardiogram (ECG) for approximately 24 h, both at the time of diagnosis and before starting any antineoplastic therapy, by calculating the average heart rate (HR) during the day, during the night, and for 24 h. For the controls, 200 patients without gastric carcinoma who belonged to the same age group as the gastric carcinoma patients were evaluated using a Holter ECG. We observed (Figure 1A,B) that during the day, higher heart rates predominated in the group of patients with gastric carcinoma than in the control group (HR during the day = 90.76 ± 13.64 beats per minute (bpm) in the group of patients with gastric carcinoma versus 82.29 ± 7.86 bpm in the control group; *p* = 0.0012). These differences were maintained overnight (HR at night = 65.74 ± 16.44 bpm in the gastric carcinoma patients versus 58.21 ± 5.11 bpm in the control group; *p* = 0.0015) and for the entire monitoring period of approximately 24 h (HR for the 24 h period = 78.25 ± 14.01 bpm in the gastric carcinoma patients versus 68.96 ± 6.80 bpm in the control group; *p* < 0.0000). Another observation was that the patients with gastric carcinoma had no significant difference in heart rate during the day compared to that during the night, as shown in patients without cancer (Figure 1C,D). All these data suggest an increased predominance of sympathetic nervous system influences in the patients with gastric carcinoma versus patients without cancer.

### 2.2. Relationship between Norepinephrine Transporter Expression and Clinicopathological Features

**Figure 2:**
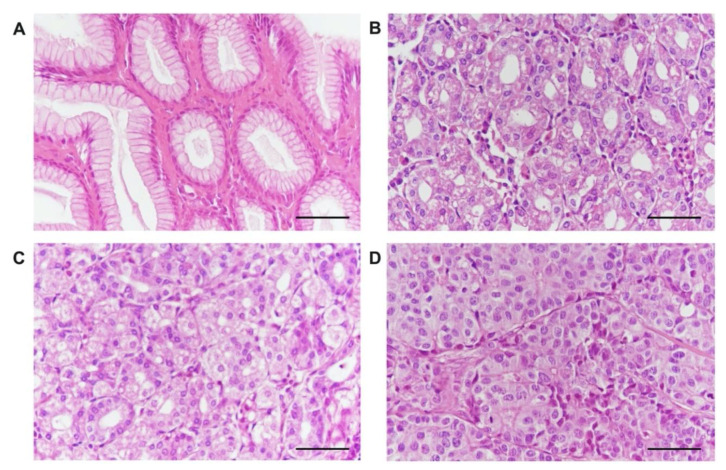
Examples of histological samples collected from the patients included in the study. (**A**) Normal gastric mucosa. (**B**) Gastric carcinoma with a high degree of cell differentiation—G1. (**C**) Gastric carcinoma with a moderate degree of cell differentiation—G2. (**D**) Gastric carcinoma with a poor degree of cell differentiation—G3. Hematoxylin and eosin staining. Magnification, 400×. Scale bars represent 20 µm.

**Figure 3:**
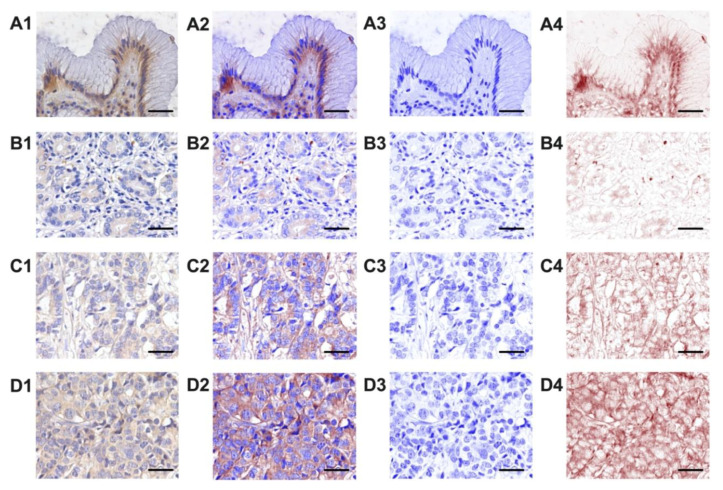
Assessment of the expression of norepinephrine transporters in normal (**A**) and tumor gastric tissue (**B**–**D**) by spectral unmixing microscopy: (**B**) well-differentiated (G1), (**C**) moderately differentiated (G2), and **(D)** poorly differentiated (G3). (1) Images from the optical microscopy; (2) slides immuno-stained for norepinephrine transporters with 3,3′-diaminobenzidines (DAB) and counterstained with hematoxylin (signals are shown overlapping); (3) images with pure hematoxylin only (nuclei and cell membranes); (4) images with pure DAB only (only the signal for norepinephrine transporters). Magnification, 400×. Scale bars represent 20 µm.

**Figure 4:**
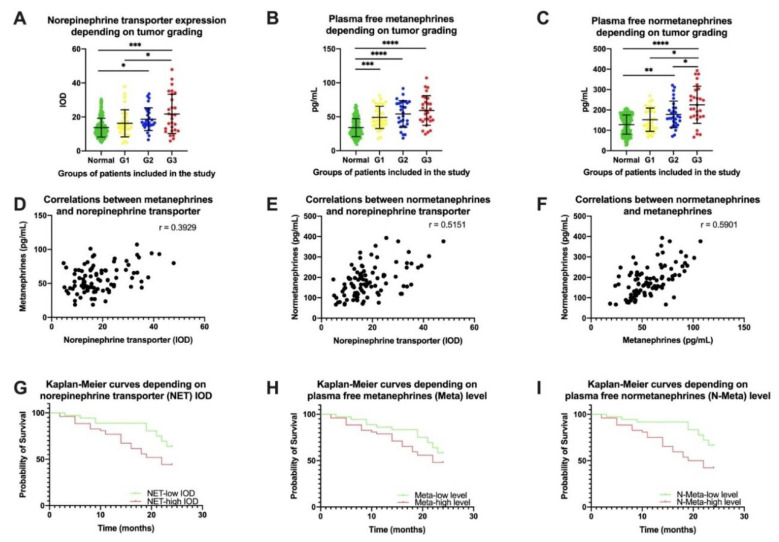
(**A**) Norepinephrine transporter expression quantified according to the integrated optical density (IOD) in cancer-free patients and in different tumor differentiation gradings in patients with gastric carcinoma. Plasma levels of free metanephrine (**B**) and normetanephrine (**C**) in cancer-free patients and in different stages of tumor differentiation in patients with gastric carcinoma. Correlations between metanephrine and norepinephrine transporter (**D**), normetanephrine and normetanephrine transporter (**E**), and normetanephrine and metanephrine (**F**). Kaplan–Meier curves depending on the norepinephrine transporter IOD (**G**), on plasma free metanephrine (**H**), and on plasma free normetanephrine (**I**). One-way ANOVA; * *p* < 0.05, ** *p* < 0.01, *** *p* < 0.001, and **** *p* < 0.0001.

**Figure 5:**
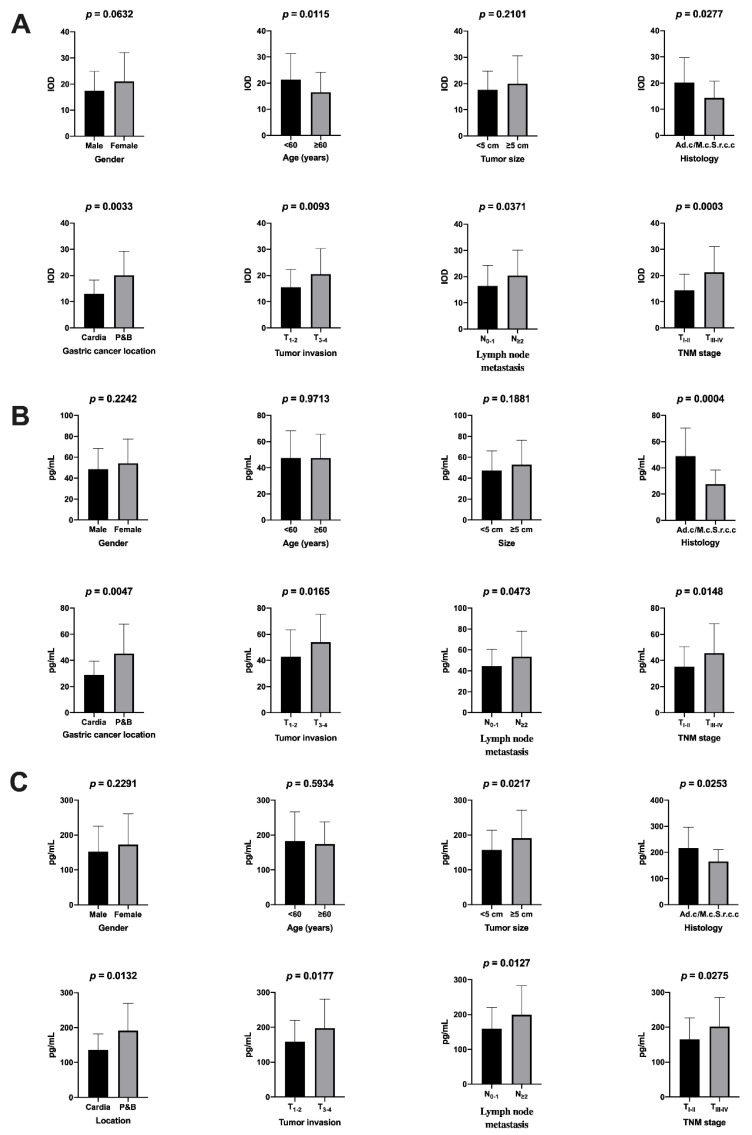
(**A**) Relationship between norepinephrine transporter expression and clinicopathological features. Relationship between plasma free metanephrine (**B**) and normetanephrine (**C**) and clinicopathological features. Ad.c., adenocarcinoma; M.c./S.r.c.c., mixed carcinoma/signet-ring-cell carcinoma; P&B, gastric body or pyloric area.

The expression of the norepinephrine transporter was analyzed in samples from patients with gastric carcinoma (N = 91) as well as from the 200 patients (controls) who required gastric resection for benign reasons (Figure 2A–D). Using multispectral microscopy (Figure 3A–D), we analyzed the expression of the norepinephrine transporter, calculating the integrated optical density (IOD) only for the target color signal. Depending on the tumor grading, we observed an increase in IOD from well-differentiated (G1) to moderately differentiated (G2) and poorly differentiated (G3) tumors (Figure 4A and Supplementary Table S1). The norepinephrine transporter expression was higher in the patients with gastric carcinoma in those aged < 60 years (*p* = 0.0115) and in those with localization of the tumor in the gastric body or pyloric area (*p* = 0.0033), with tumor invasion T_3–4_ (*p* = 0.0093), with lymph node metastasis N_≥2_ (*p* = 0.0371), and with TNM classification of malignant tumors (TNM) stages T_III–IV_ (*p* = 0.003) (Figure 5a and Supplementary Table S2).

### 2.3. Relationship between Plasma Free Metanephrine (Meta) and Normetanephrine and Clinicopathological Features

Another analysis performed in our study was measuring the free metanephrine and normetanephrine in the plasma for all the patients included before starting any therapy. Both the plasma free metanephrine and plasma free normetanephrine were higher in the patients with gastric carcinoma than those without. The cancer-free patients had plasma free metanephrine (Meta) values of 34.05 ± 13.23 pg/mL and plasma free normetanephrine (N-Meta) values of 128.3 ± 46.70 pg/mL. In the patients with gastric carcinoma, increased plasmatic levels of metanephrine and normetanephrine were correlated with tumor grading, increasing from well-differentiated (Meta = 48.09 ± 16.45 pg/mL and N-Meta = 152.1 ± 57.05 pg/mL) to moderately differentiated (Meta = 54.14 ± 19.59 pg/mL and N-Meta = 178.1 ± 65.53 pg/mL) and poorly differentiated (Meta = 59.13 ± 21.88 pg/mL and N-Meta = 225.4 ± 91.22 pg/mL) tumors (Figure 4B,C). In terms of the clinicopathological features concerned, we observed that higher free metanephrine could be found in patients with gastric carcinoma according to the histological type, i.e., adenocarcinoma (Ad.c.) as opposed to mixed carcinoma/signet-ring-cell carcinoma (M.c./S.r.c.c.) (*p* = 0.0004); the location of the tumor in the gastric body or pyloric area (*p* = 0.0047); tumor invasion T_3–4_ (*p* = 0.0165); lymph node metastasis N_≥2_ (*p* = 0.0473); and TNM stage T_III–IV_ (*p* = 0.0148) (Figure 5B and Supplementary Table S3). We also observed higher plasma free normetanephrine in patients with gastric carcinoma with a tumor size ≥5 cm (*p* = 0.0217), histological type Ad.c as opposed to M.c./S.r.c.c. (*p* = 0.0253), a location of the tumor in the gastric body or pyloric area (*p* = 0.0132), a tumor invasion T_3–4_ (*p* = 0.0177), a lymph node metastasis N_≥2_ (*p* = 0.0127), and a TNM stage T_III–IV_ (*p* = 0.0275) (Figure 5C and Supplementary Table S4). It should be noted that patients who had the signet-ring-cell gastric cancer type, despite having a low degree of differentiation (G3), had low plasma levels of free metanephrine and normetanephrine.

### 2.4. Correlation between Norepinephrine Transporter Expression and Plasma Free Metanephrine and Normetanephrine

We observed a moderate positive correlation between the IOD for norepinephrine transporter expression and plasma free metanephrine (r = 0.3929; 95% confidence interval = 0.2034–0.5540; R-squared = 0.1544), and a strong positive correlation between the IOD for norepinephrine transporter expression and plasma free normetanephrine (r = 0.5151; 95% confidence interval = 0.3459–0.6519; R-squared = 0.2654). There was also a strong positive correlation between the plasma free metanephrine and plasma free normetanephrine (r = 0.5901; 95% confidence interval = 0.4373–0.7098; R-squared = 0.3842). These data are summarized in Figure 4D–F.

### 2.5. Univariate Analysis of Prognostic Factors

The 91 patients with gastric carcinoma were divided into a low-NET group (N = 36/91) and a high-NET group (N = 55/91) based on the median IOD for the norepinephrine transporter. Depending on cut-off levels for plasma free metanephrine (65 pg/mL) and normetanephrine (196 pg/mL), the patients with gastric carcinoma were divided into low- (N = 39/91) and high-Meta groups (N = 52/91) and into low-(N = 33/91) and high-N-Meta groups (N = 58/91). Univariate analysis with a log-rank test indicated that the high-NET patients had a significantly poorer survival rate at two years after inclusion in the study than the low-NET patients (44.23% vs. 63.88%; *p* = 0.0358; hazard ratio and its reciprocal = 1.956 and 0.5140, respectively; Figure 4G). Lower survival rates were also observed in the patients with higher metanephrine but without statistical significance (48.07% vs. 58.33%; *p* = 0.1487; hazard ratio and its reciprocal = 1.462 and 0.6838, respectively; Figure 4H), as well as in those with high free normetanephrine with statistical significance (42.30% vs. 66.67%; *p* = 0.0104; hazard ratio and its reciprocal = 2.289 and 0.4369, respectively; Figure 4I).

## 3. Discussion

In this study, we analyzed the correlations between the clinicopathological aspects of patients diagnosed with gastric cancer (gastric adenocarcinoma) and the involvement of the autonomic nervous system in the carcinogenesis process by identifying certain features of the sympathetic nervous system and the norepinephrine transporter, identified in neoplastic cells. We attempted to support the claim that the vegetative nervous system can influence the development and evolution of gastric cancer.

The sympathetic nervous system is part of the vegetative nervous system and responsible for the fight reaction, also known as the sympathetic–adrenal response. It secretes adrenaline (epinephrine) and noradrenaline (norepinephrine), catecholamines that are subsequently released into the blood [15]. The action exerted by the sympathetic nervous system causes a series of reactions in various organs of the body, such as an increased heart rate, decreased motility in the large intestine, reduced secretions by salivary glands, and vasoconstriction [16]. Free plasma metanephrine and normetanephrine are metabolites of catecholamines, the latter being considered a hormone that is released into the blood, especially during periods of physical or emotional stress, depression, or anxiety, causing both psychological and endocrine changes [17]. These catecholamines produced from the precursor tyrosine can, on the one hand, alter the immune response and, on the other hand, promote several biological signaling pathways involved in tumor initiation, growth, and metastasis [16,17,18,19].

Regarding the involvement of the sympathetic nervous system in the development and evolution of gastric cancer, the primary pathway is mediated by the action of neurotransmitters on β2-adrenergic receptors, activating an intracellular signaling cascade via adenylyl cyclase [18,19]. Studies have highlighted the impact of the main neurotransmitter of the sympathetic nervous system, norepinephrine, on vascular endothelial growth factor (VEGF) and matrix metalloproteinase 2/9 (MMP-2 and MMP-9) [20]. Other studies have also shown an important role of norepinephrine in epithelial–mesenchymal transition (EMT). For example, Shan et al. demonstrated that norepinephrine causes, in gastric carcinoma, a decrease in E-cadherin expression and an increase in vimentin expression; both changes increase cell motility and confer the ability of tumor invasion [21]. This mechanism can occur through the β2-adrenergic receptor (AR)–hypoxia-inducible factor-1-alpha axis, which is also involved in the promotion of tumor progression by chronic stress in animal cancer models [22]. EMT can also be initiated in gastric cancer by the β2-AR–metalloproteinase (MMP)-7 pathway through the activation of AP-1 and signal transducer and transcriptional activator 3 (STAT3) [23,24].

We found that the highest plasma values of serum metanephrine and normetanephrine were increased in patients with poorly differentiated gastric adenocarcinoma. They varied according to the degree of differentiation, and increased values were found among those with localization in the gastric body or pyloric area and with histopathological aspects of adenocarcinoma, as well as among patients who had metastases in regional lymph nodes or distant metastasis. In this regard, a recent study that evaluated the activity of periostin, which mediates the critical steps in gastric carcinoma, showed that it is expressed in the stroma of gastric carcinoma but not in normal gastric tissue, and this is strongly correlated with the expression of alpha-smooth muscle actin (SMA) [25]. Isoprenaline causes an increase in periostin expression in gastric cancer, with the activation of the previously mentioned axis, but it can also promote angiogenesis by stimulating VEGF secretion and the upregulation of VEGFR2 and plexin-A1 [26,27].

Another aspect highlighted by our study is the implications of psychological stress for the initiation and progression of gastric carcinoma. Psychological stress initiates a response of the hypothalamic–pituitary–adrenal axis, which raises catecholamine levels; catecholamines interact with certain biological components of tumor cells through certain signaling pathways. This can lead to the progression of certain cancers, such as those of the ovaries, nasopharynx, or pancreas [27,28].

Regarding the influence of catecholamines on therapy for severe gastric cancer, it has been observed that the stimulation of gastric cancer cells with catecholamines in vitro increases trastuzumab resistance by not only activating STAT3 and extracellular signal-regulated kinases (ERKs) but also by upregulating mucin 4 (MUC4) expression [29]. However, these cellular signaling mechanisms induced by catecholamines may become possible therapeutic targets. For example, propranolol, a non-selective adrenergic blocker, can cause cell cycle arrest and induce apoptosis in gastric carcinoma cells by blocking nuclear factor-kB (NF-kB), MMP2/9, VEGF, and cyclooxygenase-2 (COX-2) [30,31].

It is well known that the sympathetic nervous system influences cardiac activity, causing an increase in heart rate, as was shown in our patients. Most of the patients with gastric cancer in our study had increased heart rates directly proportional to the plasma levels of free metanephrine and normetanephrine. Shi et al. recently reported that the severity of gastric cancer in diagnosed patients can also be predicted by perturbations in the nonlinear dynamic patterns of heart rate variability (HRV) [32].

In other primary tumors, the activity of the sympathetic nervous system has been evaluated in both preclinical and clinical studies. The plasma norepinephrine and epinephrine concentrations are significantly higher in patients with oral and oropharyngeal squamous cell carcinoma (SCC) than in non-cancer patients [33]. In epithelial ovarian cancer, norepinephrine reduces cisplatin’s efficacy and can affect DNA integrity [34]. Epinephrine increases the phosphorylation of p38 MAPK in breast cancer cells and, thereby, enhances the malignancy of this type of cancer [35]. A chemical sympathectomy markedly reduces the incidence of fibrosarcoma and significantly prolongs survival in rats [36]. Regarding other tumors of the gastrointestinal tract, it has been found that norepinephrine facilitates tumor growth in pancreatic cancer [37], induces hepatocellular carcinoma invasion and anoikis resistance through β2-AR-mediated epidermal growth factor receptor transactivation [38], and facilitates cell proliferation in esophageal squamous cell carcinoma [39]. Catecholamines have also been shown to promote metastasis and tumor progression in prostate and lung cancers and melanoma [40].

## 4. Materials and Methods

### 4.1. Patients

**Figure 6:**
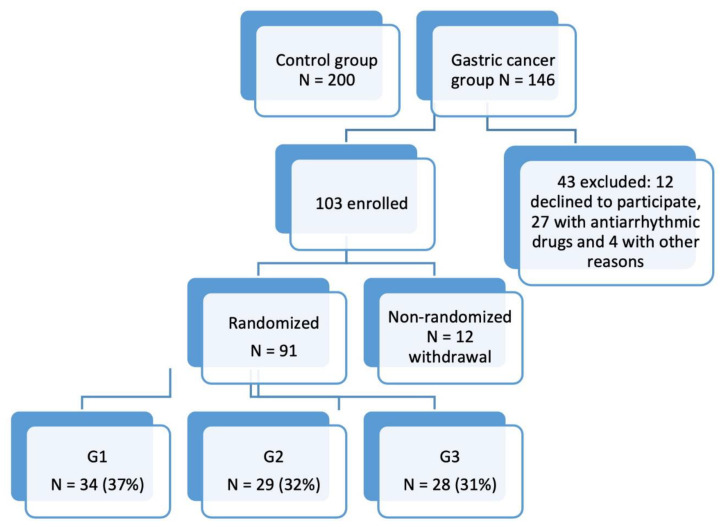
Design of the study.

This was an observational study, in which 91 patients diagnosed with gastric carcinoma with different degrees of tumor differentiation were consecutively included, following surgery or upper digestive endoscopy, at the Emergency County Hospital of Craiova, Romania, between November 2017 and October 2018. For the controls, we delimited a group of 200 patients without cancer of the same age group and gender as the cancer patients. We set the primary endpoint as mortality from any cause in the first two years after enrolling in the study. The design of the study is shown in Figure 6.

All of the stages of the study were explained to the patients before commencement, and participation was possible only after providing written consent, with the patients being informed about the confidentiality of personal data and the procedures being performed in accordance with current regulations, without negatively influencing normal diagnostic or treatment procedures.

This study was approved by the Ethics Committee of the University of Medicine and Pharmacy of Craiova (No. 71/02.04.2017), respecting the ethical principles underlying the Declaration of Helsinki and the University Code of Ethics on Good Conduct of Research and the codes of practice established by the Code of Medical Ethics.

Patients diagnosed with gastric cancer, confirmed by postoperative histopathological examination or biopsy, had blood samples taken for the measurement of serum metanephrine and normetanephrine, after which they underwent Holter ECGs, before starting chemotherapeutic treatment, to avoid possible post-drug interactions.

### 4.2. Assessment of Heart Rate

To assess the predominance of the sympathetic or parasympathetic nervous system, the patients were also monitored using a Holter ECG TLC5000 (Contec Medical Systems, Qinhuangdao, Hebei Province, China), together with a former analysis of heart rate variability in terms of both frequency and the field of time. Ten patch electrodes were applied to the patients’ chests, through which 12 ECG leads were connected. The important parameters in our study were the minimum, average, and maximum heart rate, not only over 24 h but also during the night and day. The ECG Holter parameters were interpreted according to the recommendations of the American College of Cardiology/American Heart Association (ACC/AHA) ambulatory electrocardiography guide [41]. It should be noted that the patients were examined by this method after the diagnosis of gastric adenocarcinoma was confirmed. Another important criterion was a lack of previous medication that could have influenced the heart rhythm (especially betablockers, calcium channel blockers, current blockers, or other antiarrhythmics). All the patients in our study performed at least 150 min of moderate-intensity aerobic physical activity per week. There were no differences between the groups included in the study regarding the degree of physical activity. Since all the patients underwent gastrectomy and since this intervention involved damage to local nerve plexuses, we unfortunately did not consider it appropriate to analyze the heart rate variability postoperatively. Moreover, postoperative stress is another factor that inevitably changes heart rate variability.

### 4.3. Histopathological Examination

A histopathological examination was performed following surgery or tumor biopsy via upper digestive endoscopy. The biological samples were introduced into 10% formic aldehyde solution, in which a neutral pH was created by adding calcium bicarbonate to neutralize the formic acid for fixation. After fixation, the biological samples were washed with water and then paraffin embedded as follows. The samples were completely dehydrated by passing them through ethyl alcohol of different concentrations. They were then clarified by removing the alcohol from the tissue, and the samples were passed through successive paraffin baths, incorporated into paraffin blocks, and solidified. Finally, the paraffin blocks were sectioned, then the sectioned tissues were glued to slides and stained with hematoxylin–eosin, after which histopathological diagnoses were established with certainty.

### 4.4. Immunohistochemistry

The paraffin-embedded tissues were sectioned into 3 μm serial sections using the HM350 rotary microtome (Thermo Fisher Scientific, Waltham, MA, USA) equipped with a section transfer system in a cold-water bath with a Peltier cooling mode. These slides were then transferred to a water bath heated to 40 °C to be stretched and evened out. The slides were recovered from the bath on blades covered with poly-lysine with a positively charged amino acid residue to increase the adhesion of the sections on the blades. The poly-lysine-coated slides were dried in a thermostat at 37 °C for 24 h.

The next day, the slides were first deparaffined by passing them through three successive xylene baths, for 15 min each, and then rehydrated in alcohol solutions with decreasing concentrations; any final traces of alcohol were removed by washing with distilled water. For antigenic recovery, the slides were boiled for 21 min in successive cycles of 3 min each, in a microwave oven, at a power of 600 W, in a solution of sodium citrate at a pH of 6. This was followed by cooling the slides for 30 min and then washing them with tap water and distilled water for 15 min. To block endogenous peroxidase activity, the slides were incubated in 1% hydrogen peroxide and distilled water for 30 min at room temperature, stored for another 30 min in 3% skimmed-milk powder in phosphate-buffered saline (PBS), and then incubated with primary antibodies at 4 °C for 18 h.

The primary antibodies used in this work were norepinephrine transporter monoclonal antibody (CL3063)/NBP2-62704 (dilution 1:20; Novus Biological, Abingdon, UK).

Finally, the signal was identified via 3,3′-diaminobenzidines (DAB) (Dako, Glostrup, Denmark). Subsequently, the slides were cover-slipped in DPX (Sigma-Aldrich, St. Louis, MO, USA) after hematoxylin and eosin staining. All the slides stained for each primary antibody were processed at the same time to observe the protocol correlation, along with the control sections, which were stained with either DAB or hematoxylin–eosin in order to obtain the pure spectrum for those colors. Negative controls were obtained by omitting primary antibodies.

### 4.5. Acquisition and Image Processing

For the quantification of the target immunohistochemical signal, and taking into account the histopathological aspect, light microscopy images were obtained using a Nikon Eclipse 90i motorized microscope (Apidrag, Bucharest, Romania). This microscope was equipped with a Nuance FX multispectral camera as well as the Nuance imaging analysis software (Perkin Elmer, Hopkinton, MA, USA). An optical microscopy image was initially obtained, followed by a mixed image (the color spectra for hematoxylin and DAB were separately superimposed on this image). In another step, separate images were obtained for hematoxylin and DAB (Supplementary Figure S1). The unmixed DAB signal was quantified by randomly obtaining 10 images captured with a 20× lens. The color signal was quantitatively analyzed based on the integrated optical density, using the Image-Pro Plus AMS 7 image analysis software (Media Cybernetics, Bethesda, MD, USA). With the help of this software, regions of interest were defined where we evaluated the color signals and calculated the IOD, while the stroma was manually excluded from the obtained images.

### 4.6. Dosage of Plasma Free Metanephrine and Normetanephrine

Blood samples were taken from fasted patients who had avoided alcohol and caffeine for 24 h before sampling. They were also informed to avoid certain drugs that may influence serum metanephrine and normetanephrine, such as acetaminophen, tricyclic antidepressants, phenoxybenzamine, alpha-agonists, or monoaminoxidase inhibitors. For patients undergoing treatment with these drugs, their medication was discontinued for at least five days before sampling.

Venous blood was collected in a pre-cooled ethylenediaminetetraacetic acid (EDTA) K3 vacutainer, which was then gently shaken, overturned, and placed on ice; it was transported to the laboratory within 2 h. The samples were processed no later than 2 h after sampling.

The serum metanephrine and normetanephrine were quantified via the competitive enzyme-linked immunosorbent assay (ELISA), and the values were determined after a precipitation stage. Their reference values were the following: metanephrine, <65 pg/mL, and normetanephrine, <196 pg/mL, with detection limits of 5 and 10 pg/mL, respectively [42].

### 4.7. Statistical Analysis

The data obtained with the Image-Pro Plus AMS 7 image analysis software were exported to Microsoft Excel 2010 (Microsoft Corporation, Redmond, WA, USA) and analyzed using GraphPad Prism 8 (San Diego, CA, USA). All the results are reported as the means and standard deviations. To compare the means of two groups, we used Student’s *t*-test. To compare the means of more than two groups, we used an ANOVA. To examine the correlations between the different categories of data, we used Pearson’s correlation test. To analyze whether there was a link between a variable and survival time, we used the log-rank test. *p* < 0.05 was considered to indicate a statistically significant difference between the compared means from the various groups.

## 5. Conclusions

The predominance of the sympathetic nervous system’s activity in patients with gastric cancer, through increased heart rates, elevated plasma free metanephrine and normetanephrine, and increased expression of the norepinephrine transporter in tumor cells, is a negative prognostic factor for these patients. These observations may highlight future therapeutic or prognostic targets.

## Supplementary Materials

The supplementary materials can be found at https://www.mdpi.com/1422-0067/22/4/2042/s1: Figure S1, example of spectral unmixing for the series of slides; Table S1, norepinephrine transporter expression quantified according to the integrated optical density in cancer-free patients and in different tumor differentiation gradings in patients with gastric carcinoma; Table S2, norepinephrine transporter expression quantified depending on clinicopathological features; Table S3, plasma free metanephrine level (pg/mL) depending on the clinicopathological features; Table S4, plasma free normetanephrine level (pg/mL) depending on the clinicopathological features.

## Abbreviations

bpm	Beats per minute
DAB	3,3′-diaminobenzidine
EDTA	Ethylenediaminetetraacetic acid
ELISA	Enzyme-linked immunosorbent assay
HRV	Heart rate variability
IOD	Integrated optical density
Meta	Metanephrine
N-meta	Normetanephrine
NET	Norepinephrine transporter
STAT3	Signal transducer and activator of transcription 3
VEGF	Vascular endothelial growth factor
VEGFR	Vascular endothelial growth factor receptor

## References

[1] Global Cancer Observatory. https://gco.iarc.fr/today/online-analysis-pie?v=2018&mode=cancer&mode_population=continents&population=900&populations=900&key=total&sex=0&cancer=39&type=0&statistic=5&prevalence=0&population_group=0&ages_group%5B%5D=0&ages_group%5B%5D=17&nb_items=7&group_cancer=1&include_nmsc=1&include_nmsc_other=1&half_pie=0&donut=0&population_group_globocan_id=.

[2] Kim J., Cho Y.A., Choi W.J., Jeong S.H. (2014). Gene-diet interactions in gastric cancer risk: A systematic review. World J. Gastroenterol..

[3] Entschladen F., Palm D., Lang K., Drell IV T.L., Zaenker K.S. (2006). Neoneurogenesis: Tumors may initiate their own innervation by the release of neurotrophic factors in analogy to lymphangiogenesis and neoangiogenesis. Med. Hypotheses.

[4] Gao J.P., Xu W., Liu W.T., Yan M., Zhu Z.G. (2018). Tumor heterogeneity of gastric cancer: From the perspective of tumor-initiating cell. World J. Gastroenterol..

[5] Entschladen F., Palm D., Niggemann B., Zaenker K.S. (2008). The cancer’s nervous tooth: Considering the neruronal crosstalk within tumors. Semin. Cancer Biol..

[6] Schuller H.M., Al-Wadei H.A., Majidi M. (2008). GABA B receptor is a novel drug target for pancreatic cancer. Cancer.

[7] Batsakis J.G. (1985). Nerves and neurotropic carcinomas. Ann. Otol. Rhinol. Laryngol..

[8] Rodin A.E., Larson D.L., Roberts D.K. (1967). Nature of the perineural space invaded by prostatic carcinoma. Cancer.

[9] Anderson P.R., Hanlon A.L., Patchefsky A., Al-Saleem T., Hanks G.E. (1998). Perineural invasion and Gleason 7–10 tumors predict increased failure in prostate cancer patients with pretreatment PSA. Int. J. Radiat. Oncol. Biol. Phys..

[10] Schroeter S., Apparsundaram S., Wiley R.G., Miner L.H., Sesack S.R., Blakely R.D. (2000). Immunolocalization of the cocaine- and antidepressant-sensitive l-norepinephrine transporter. J. Comp. Neurol..

[11] Tellioglu T., Robertson D. (2001). Genetic or acquired deficits in the norepinephrine transporter: Current understanding of clinical implications. Expert Rev. Mol. Med..

[12] Kristensen A.S., Andersen J., Jørgensen T.N., Sørensen L., Eriksen J., Loland C.J., Strømgaard K., Gether U. (2011). SLC6 neurotransmitter transporters. Structure, function and regulation. Pharmacol. Rev..

[13] Torres G.E., Gainetdinov R.R., Caron M.G. (2003). Plasma membrane monoamine transporters: Structure, regulation and function. Nat. Rev. Neurosci..

[14] Browning K.N., Travagli R.A. (2014). Central nervous system control of gastrointestinal motility and secretion and modulation of gastrointestinal functions. Compr. Physiol..

[15] Wang K., Zhao X.H., Liu J., Zhang R., Li J.P. (2020). Nervous system and gastric cancer. Biochim. Biophys. Acta (BBA) Rev. Cancer.

[16] Repasky E.A., Eng J., Hylander B.L. (2015). Stress, metabolism and cancer: Integrated pathways contributing to immune suppression. Cancer J. (Sudbury Mass.).

[17] Peters L.J., Kelly H. (1977). The influence of stress and stress hormones on the transplantability of a non-immunogenic syngeneic murine tumor. Cancer.

[18] Lin X., Luo K., Lv Z., Huang J. (2012). Beta-adrenoceptor action on pancreatic cancer cell proliferation and tumor growth in mice. Hepato Gastroenterol..

[19] Ciurea R.N., Rogoveanu I., Pirici D., Târtea G.C., Streba C.T., Florescu C., Cătălin B., Puiu I., Târtea E.A., Vere C.C. (2017). B2 adrenergic receptors and morphological changes of the enteric nervous system in colorectal adenocarcinoma. World J. Gastroenterol..

[20] Yang E.V., Sood A.K., Chen M., Li Y., Eubank T.D., Marsh C.B., Jewell S., Flavahan N.A., Morrison C., Yeh P.E. (2006). Norepinephrine up-regulates the expression of vascular endothelial growth factor, matrix metalloproteinase (MMP)-2, and MMP-9 in nasopharyngeal carcinoma tumor cells. Cancer Res..

[21] Shan T., Cui X., Li W., Lin W., Li Y., Chen X., Wu T. (2014). Novel regulatory program for norepinephrine-induced epithelial-mesenchymal transition in gastric adenocarcinoma cell lines. Cancer Sci..

[22] Hu H.T., Ma Q.Y., Zhang D., Shen S.G., Han L., Ma Y.D., Li R.F., Xie K.P. (2010). HIF-1alpha links beta-adrenoceptor agonists and pancreatic cancer cells under normoxic condition. Acta Pharmacol. Sin..

[23] Shi M., Liu D., Duan H., Han C., Wei B., Qian L., Chen C., Guo L., Hu M., Yu M. (2010). Catecholamine up-regulates MMP-7 expression by activating AP-1 and STAT3 in gastric cancer. Mol. Cancer.

[24] Lu Y.J., Geng Z.J., Sun X.Y., Li Y.H., Fu X.B., Zhao X.Y., Wei B. (2015). Isoprenaline induces epithelial-mesenchymal transition in gastric cancer cells. Mol. Cell. Biochem..

[25] Liu G.X., Xi H.Q., Sun X.Y., Geng Z.J., Yang S.W., Lu Y.J., Wei B., Chen L. (2016). Isoprenaline Induces Periostin Expression in Gastric Cancer. Yonsei Med. J..

[26] Lu Y., Xu Q., Zuo Y., Liu L., Liu S., Chen L., Wang K., Lei Y., Zhao X., Li Y. (2017). Isoprenaline/β2-AR activates Plexin-A1/VEGFR2 signals via VEGF secretion in gastric cancer cells to promote tumor angiogenesis. BMC Cancer.

[27] Lutgendorf S.K., Lamkin D.M., Jennings N.B., Arevalo J.M., Penedo F., DeGeest K., Langley R.R., Lucci J.A., Cole S.W., Lubaroff D.M. (2008). Biobehavioral influences on matrix metalloproteinase expression in ovarian carcinoma. Clin. Cancer Res..

[28] Sood A.K., Armaiz-Pena G.N., Halder J., Nick A.M., Stone R.L., Hu W., Carroll A.R., Spannuth W.A., Deavers M.T., Allen J.K. (2010). Adrenergic modulation of focal adhesion kinase protects human ovarian cancer cells from anoikis. J. Clin. Investig..

[29] Shi M., Yang Z., Hu M., Liu D., Hu Y., Qian L., Zhang W., Chen H., Guo L., Yu M. (2013). Catecholamine-Induced β2-adrenergic receptor activation mediates desensitization of gastric cancer cells to trastuzumab by upregulating MUC4 expression. J. Immunol..

[30] Liao X., Che X., Zhao W., Zhang D., Bi T., Wang G. (2010). The β-adrenoceptor antagonist, propranolol, induces human gastric cancer cell apoptosis and cell cycle arrest via inhibiting nuclear factor κB signaling. Oncol. rep..

[31] Takahashi K., Kaira K., Shimizu A., Sato T., Takahashi N., Ogawa H., Yoshinari D., Yokobori T., Asao T., Takeyoshi I. (2016). Clinical significance of β2-adrenergic receptor expression in patients with surgically resected gastric adenocarcinoma. Tumour Biol. J. Int. Soc. Oncodev. Biol. Med..

[32] Shi B., Wang L., Yan C., Chen D., Liu M., Li P. (2019). Nonlinear heart rate variability biomarkers for gastric cancer severity: A pilot study. Sci. Rep..

[33] Bastos D.B., Sarafim-Silva B., Sundefeld M., Ribeiro A.A., Brandão J., Biasoli É.R., Miyahara G.I., Casarini D.E., Bernabé D.G. (2018). Circulating catecholamines are associated with biobehavioral factors and anxiety symptoms in head and neck cancer patients. PLoS ONE.

[34] Lamboy-Caraballo R., Ortiz-Sanchez C., Acevedo-Santiago A., Matta J., NA Monteiro A., N Armaiz-Pena G. (2020). Norepinephrine-Induced DNA Damage in Ovarian Cancer Cells. Int. J. Mol. Sci..

[35] Ouyang X., Zhu Z., Yang C., Wang L., Ding G., Jiang F. (2019). Epinephrine increases malignancy of breast cancer through p38 MAPK signaling pathway in depressive disorders. Int. J. Clin. Exp. Pathol..

[36] Lackovicova L., Banovska L., Bundzikova J., Janega P., Bizik J., Kiss A., Mravec B. (2011). Chemical sympathectomy suppresses fibrosarcoma development and improves survival of tumor-bearing rats. Neoplasma.

[37] Renz B.W., Takahashi R., Tanaka T., Macchini M., Hayakawa Y., Dantes Z., Maurer H.C., Chen X., Jiang Z., Westphalen C.B. (2018). β2 Adrenergic-Neurotrophin Feedforward Loop Promotes Pancreatic Cancer. Cancer Cell.

[38] Li J., Yang X.M., Wang Y.H., Feng M.X., Liu X.J., Zhang Y.L., Huang S., Wu Z., Xue F., Qin W.X. (2014). Monoamine oxidase A suppresses hepatocellular carcinoma metastasis by inhibiting the adrenergic system and its transactivation of EGFR signaling. J. Hepatol..

[39] Liu X., Wu W.K., Yu L., Sung J.J., Srivastava G., Zhang S.T., Cho C.H. (2008). Epinephrine stimulates esophageal squamous-cell carcinoma cell proliferation via beta-adrenoceptor-dependent transactivation of extracellular signal-regulated kinase/cyclooxygenase-2 pathway. J. Cell. Biochem..

[40] Jiang S.H., Hu L.P., Wang X., Li J., Zhang Z.G. (2020). Neurotransmitters: Emerging targets in cancer. Oncogene.

[41] Crawford M.H., Bernstein S.J., Deedwania P.C., DiMarco J.P., Ferrick K.J., Garson A., Green. L.A., Greene H.L., Silka M.J., Stone P.H. (1999). ACC/AHA guidelines for ambulatory electrocardiography: Executive summary and recommendations. A report of the American College of Cardiology/American Heart Association task force on practice guidelines (committee to revise the guidelines for ambulatory electrocardiography). Circulation.

[42] Eisenhofer G., Peitzsch M. (2014). Laboratory evaluation of pheochromocytoma and paraganglioma. Clin. Chem..

