# Supplementary material for: Assessment of Epinephrine and Norepinephrine in Gastric Carcinoma
Source: Int J Mol Sci. 2021 Feb 18;22(4):2042. doi: 10.3390/ijms22042042 (PMC7922341; doi:10.3390/ijms22042042)
Supplement: Supplementary file 1 [file ijms-22-02042-s001.zip › AMM_et_al.Supp/Supp Table S1.docx]

| Tissue | Normal | G1 | G2 | G3 |
| --- | --- | --- | --- | --- |
| Number of values | 200 | 34 | 29 | 28 |
| Minimum | 5,020 | 4,670 | 6,560 | 5,890 |
| Maximum | 30,46 | 37,89 | 33,58 | 47,85 |
| Range | 25,44 | 33,22 | 27,02 | 41,96 |
| Mean | 13,71 | 16,27 | 18,67 | 21,79 |
| Std. Deviation | 5,524 | 7,980 | 6,697 | 11,52 |
| Std. Error of Mean | 0,3906 | 1,369 | 1,244 | 2,177 |

**Supplementary Table S1.** Norepinephrine transporter expression quantified by the integrated optical density (IOD) in cancer-free patients and in different tumor differentiation grading in patients with gastric carcinoma.
