# Supplementary material for: Assessment of Epinephrine and Norepinephrine in Gastric Carcinoma
Source: Int J Mol Sci. 2021 Feb 18;22(4):2042. doi: 10.3390/ijms22042042 (PMC7922341; doi:10.3390/ijms22042042)
Supplement: Supplementary file 1 [file ijms-22-02042-s001.zip › AMM_et_al.Supp/Supp Table S3.docx]

**Supplementary Table S3.** Plasma free metanephrines level (pg/mL) depending on clinicopathological features.

| Clinicopathological features | | n. | Metanephrines level (pg/mL)  Mean$\pm$St.dev. | P-value |
| --- | --- | --- | --- | --- |
| Gender | Male | 57 | 48,69±19,76 | 0,2242 |
|  | Female | 34 | 54,27±23,11 |  |
| Age group | <60 | 42 | 47,56±20,86 | 0,9713 |
|  | ≥60 | 49 | 47,41±18,28 |  |
| Tumor size | <5 cm | 47 | 47,18±18,75 | 0,1881 |
|  | ≥5 cm | 44 | 53,06±23,40 |  |
| Histology | Adenocarcinoma | 77 | 48,96±21,32 | 0,0004 |
|  | Mixed carcinoma/Signet ring cell carcinoma | 14 | 27,53±10,91 |  |
| Location | Cardia | 17 | 28,75±10,57 | 0,0047 |
|  | Gastric body or pyloric area | 74 | 45,17±45,17 |  |
| Tumor invasion | T_1-2_ | 36 | 42,98±20,55 | 0,0165 |
|  | T_3-4_ | 55 | 53,98±21,27 |  |
| Lymph node  metastasis | N_0-1_ | 39 | 44,59±16,16 | 0,0473 |
|  | N_≥2_ | 52 | 53,66±24,41 |  |
| TNM  stage | T_I-II_ | 38 | 35,16±15,33 | 0,0148 |
|  | T_III-IV_ | 53 | 45,62±22,43 |  |
