# Supplementary material for: Assessment of Epinephrine and Norepinephrine in Gastric Carcinoma
Source: Int J Mol Sci. 2021 Feb 18;22(4):2042. doi: 10.3390/ijms22042042 (PMC7922341; doi:10.3390/ijms22042042)
Supplement: Supplementary file 1 [file ijms-22-02042-s001.zip › AMM_et_al.Supp/Supp Table S2.docx]

**Supplementary Table S2.** Norepinephrine transporter expression quantified by the integrated optical density (IOD) depending on clinicopathological features.

| Clinicopathological features | | n. | Norepinefrine transporter IOD  Mean$\pm$St.dev. | P-value |
| --- | --- | --- | --- | --- |
| Gender | Male | 57 | 17,37±7,47 | 0,0632 |
|  | Female | 34 | 21,02±11,00 |  |
| Age group | <60 | 42 | 21,30±9,95 | 0,0115 |
|  | ≥60 | 49 | 16,53±7,68 |  |
| Tumor size | <5 cm | 47 | 17,57±7,22 | 0,2101 |
|  | ≥5 cm | 44 | 19,97±10,65 |  |
| Histology | Adenocarcinoma | 77 | 20,25±9,46 | 0,0277 |
|  | Mixed carcinoma/Signet ring cell carcinoma | 14 | 14,35±6,33 |  |
| Location | Cardia | 17 | 12.,99±5,28 | 0,0033 |
|  | Gastric body or pyloric area | 74 | 20,05±9,27 |  |
| Tumor invasion | T_1-2_ | 36 | 15,56±6,82 | 0,0093 |
|  | T_3-4_ | 55 | 20,53±9,76 |  |
| Lymph node  metastasis | N_0-1_ | 39 | 16,45 ±7,79 | 0,0371 |
|  | N_≥2_ | 52 | 20,44 ±9,64 |  |
| TNM  stage | T_I-II_ | 38 | 14,33 ±6,21 | 0,0003 |
|  | T_III-IV_ | 53 | 21,22±9,85 |  |
