# Supplementary material for: Assessment of Epinephrine and Norepinephrine in Gastric Carcinoma
Source: Int J Mol Sci. 2021 Feb 18;22(4):2042. doi: 10.3390/ijms22042042 (PMC7922341; doi:10.3390/ijms22042042)

**Supplementary FigureS1.** Example of spectral unmixing for the series of slides. A: Immunostained for NET with DAB and counterstained with hematoxylin; B: Pure DAB and hematoxylin signals are shown either overlapping or (C and D), individually.


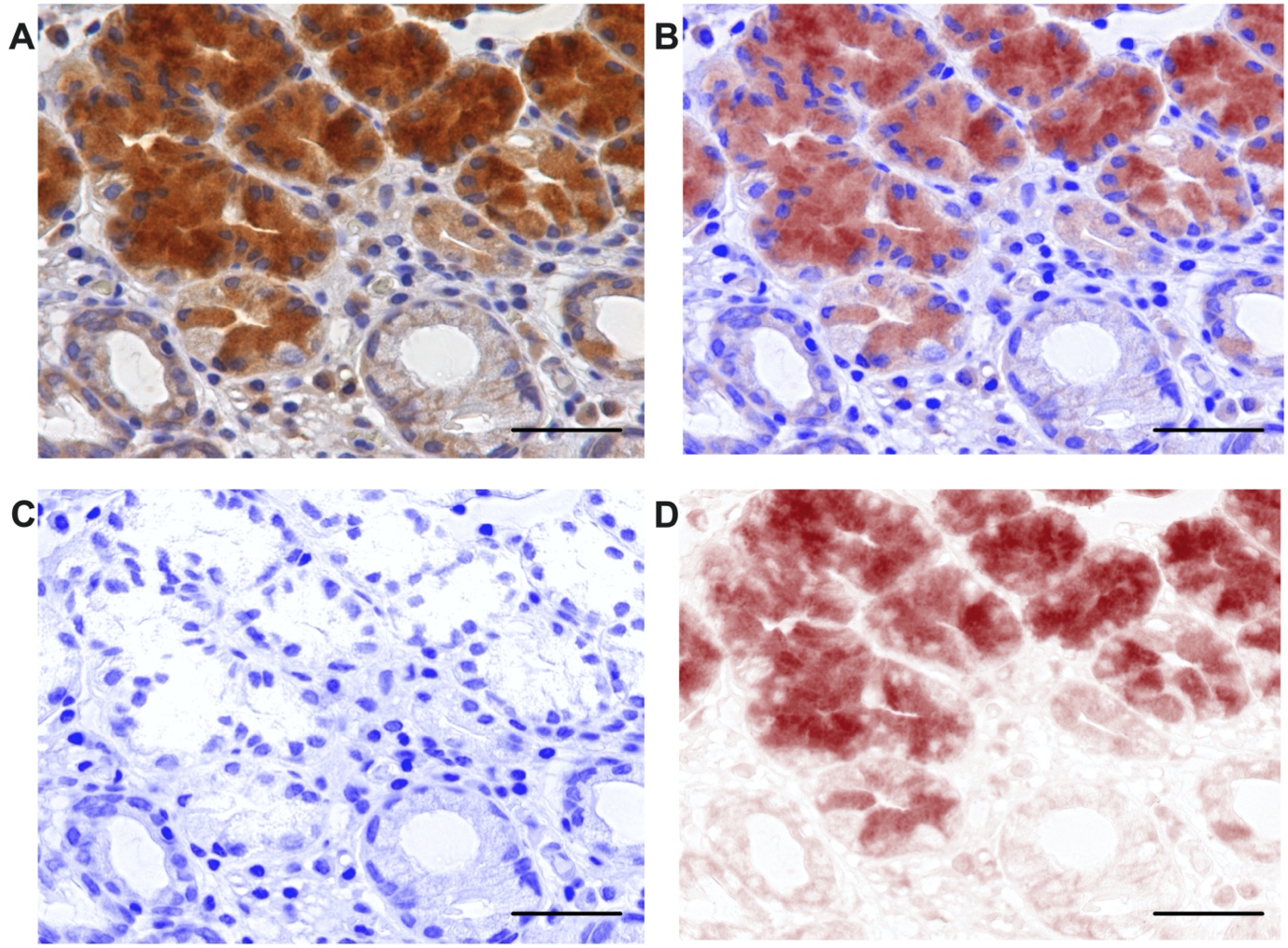

Supplement: Supplementary file 1 [file ijms-22-02042-s001.zip › AMM_et_al.Supp/Supp Figure S1.docx]
