# Supplementary material for: Assessment of Epinephrine and Norepinephrine in Gastric Carcinoma
Source: Int J Mol Sci. 2021 Feb 18;22(4):2042. doi: 10.3390/ijms22042042 (PMC7922341; doi:10.3390/ijms22042042)
Supplement: Supplementary file 1 [file ijms-22-02042-s001.zip › AMM_et_al.Supp/Supp Table S4.docx]

**Supplementary Table S4.** Plasma free normetanephrines level (pg/mL) depending on clinicopathological features.

| Clinicopathological features | | n. | Normetanephrines level (pg/mL)  Mean$\pm$St.dev. | P-value |
| --- | --- | --- | --- | --- |
| Gender | Male | 57 | 152,2±72,89 | 0,2291 |
|  | Female | 34 | 172,9±87,73 |  |
| Age group | <60 | 42 | 182,3±84,51 | 0,5934 |
|  | ≥60 | 49 | 173,9±64,33 |  |
| Tumor size | <5 cm | 47 | 156,9±56,88 | 0,0217 |
|  | ≥5 cm | 44 | 190,8±80,51 |  |
| Histology | Adenocarcinoma | 77 | 216,0±80,22 | 0,0253 |
|  | Mixed carcinoma/Signet ring cell carcinoma | 14 | 165,7±45,10 |  |
| Location | Cardia | 17 | 136,4±45,20 | 0,0132 |
|  | Gastric body or pyloric area | 74 | 191,3±78,49 |  |
| Tumor invasion | T_1-2_ | 36 | 158,8±61,56 | 0,0177 |
|  | T_3-4_ | 55 | 197,7±82,93 |  |
| Lymph node  metastasis | N_0-1_ | 39 | 159,3±61,35 | 0,0127 |
|  | N_≥2_ | 52 | 199,4±82,94 |  |
| TNM  stage | T_I-II_ | 38 | 165,6±61,63 | 0,0275 |
|  | T_III-IV_ | 53 | 201,6±84,05 |  |
